# A DNA test for high incidence of soft scald and soggy breakdown postharvest disorders in *Malus domestica* Borkh

**DOI:** 10.1007/s11032-021-01245-w

**Published:** 2021-09-11

**Authors:** Baylee A. Miller, John R. Tillman, Nicholas P. Howard, Sarah A. Kostick, Kate M. Evans, James J. Luby

**Affiliations:** 1 Department of Horticultural Science, University of Minnesota, St Paul, MN 55108 USA; 2 Fresh Forward Breeding and Marketing, Eck en Wiel, the Netherlands; 3 Plant Breeding, Wageningen University and Research, Wageningen, the Netherlands; 4 Tree Fruit Research and Extension Center, Department of Horticulture, Washington State University, Wenatchee, WA 98801 USA

**Keywords:** Apple, Kompetitive Allele Specific PCR, KASP™ marker, Single nucleotide polymorphism, SNP, Marker-assisted breeding

## Abstract

The ‘Honeycrisp’ apple, an economically important cultivar and breeding parent, is prone to soft scald and soggy breakdown postharvest physiological disorders. Phenotypic evaluation of soft scald is time consuming and costly, making it an excellent target for DNA-informed breeding. The objective of this study was to develop a DNA test for a soft scald and soggy breakdown quantitative trait locus (QTL) on linkage group two (LG2) that was characterized in a previous study. ‘Honeycrisp’ is homozygous for the undesirable high disorder incidence haplotype (HDI) at this QTL. In this study, sixteen single nucleotide polymorphism markers were evaluated for their associations with the HDI haplotype in a set of 132 unique cultivars and important breeding parents. A DNA test was successfully developed utilizing KASP™ (Kompetitive Allele Specific PCR) chemistry to identify the number of HDI haplotypes in individuals. This test had a 100% accuracy for detecting homozygous unfavorable HDI individuals and has an expected 88% accuracy over all three haplotype copy groups across the evaluated germplasm. This DNA test is a promising tool for minimizing the chances of selecting individuals that exhibit high incidence of soft scald postharvest disorder in ‘Honeycrisp’-related germplasm.

**Supplementary Information:**

The online version contains supplementary material available at 10.1007/s11032-021-01245-w.

## Introduction

The ‘Honeycrisp’ apple, an economically important cultivar and breeding parent, is prone to soft scald and soggy breakdown postharvest physiological disorders. Soft scald and soggy breakdown become evident in fruit only after they have been in cold storage for several weeks, rendering the fruit unmarketable due to browning of flesh and skin (Brooks and Harley 1934). ‘Honeycrisp’ fruit often exhibit very high incidence of these disorders under certain storage conditions and growing environments, causing a significant loss of marketable fruit. Various cold storage regimes have been suggested to reduce soft scald incidence. For example, Watkins and Rosenberger (2012) found that soft scald and soggy breakdown could be significantly minimized when ‘Honeycrisp’ fruit were treated with a conditioning period before placing fruit into cold storage. Controlled atmosphere (CA) cold storage and treatment with diphenylamine (DPA) also reduced soft scald and soggy breakdown incidence in ‘Honeycrisp’ (Watkins et al. 2004). However, these practices do not eliminate soft scald and optimal storage conditions vary by apple cultivar, requiring separate storage accommodations (Watkins and Nock 2003) as well as costly and time-consuming research for each new apple cultivar. Additionally, not all producers have the resources required to store individual cultivars in unique storage conditions. Breeding new cultivars that are less prone to soft scald and soggy breakdown might be a more effective long-term solution.

A large effect QTL on linkage group two (LG2) associated with variation in incidence of soft scald in University of Minnesota (UMN) pedigree-connected apple breeding germplasm was reported by Howard et al. (2018). Howard et al. (2018) identified a high disorder incidence (HDI) haplotype associated with high relative soft scald incidence at the LG2 QTL. The HDI haplotype was reported to have an additive effect, with two copies associated with the highest disorder incidence, one copy associated with moderate incidence, and zero copies associated with low relative incidence. ‘Honeycrisp’ is homozygous for the HDI haplotype at this locus; therefore, inheritance of one HDI haplotype is unavoidable when using ‘Honeycrisp’ as a parent. Additionally, several other cultivars (e.g., ‘Ambrosia,’ ‘Cripps Pink,’ ‘Golden Delicious,’ ‘Honeygold,’ and ‘MonArk’) have one HDI haplotype (Howard et al. 2018).

Apple breeding efforts are now widely utilizing ‘Honeycrisp’ as a breeding parent. With many recent cultivar releases having ‘Honeycrisp’ as a parent, including ‘Minneiska’ (SweeTango®), ‘MN55’ (First Kiss® and Rave®), ‘WA 38’ (Cosmic Crisp®) ‘MAIA1’ (EverCrisp®), and ‘New York 1’ (SnapDragon®) (Bedford and Luby 2008, 2016; Brown and Maloney 2011; Dodd et al. 2014; Evans et al. 2012), one copy of the HDI haplotype is inherited into offspring from ‘Honeycrisp’ and an additional copy might be inherited from other parents. Evaluation of fruit is a time-consuming and resource-intensive process in apple breeding programs. The fruit evaluation process requires breeders to maintain large seedling orchards for many years and most seedlings demonstrate undesirable traits that often cannot be identified until fruit are evaluated. The long juvenility period for apple trees and necessity of sufficient fruit for storage trials render postharvest disorders to be among the last traits to be evaluated and characterized for potential new cultivars. A DNA test that could be used to identify individuals with one or more unfavorable HDI haplotypes at the LG2 QTL would therefore be beneficial in reducing the chances of identifying a promising new selection only to later identify problematic storage disorders. However, to the best of the authors’ knowledge, no DNA test has been reported for the soft scald and soggy breakdown LG2 QTL.

The objective of this study was to develop and validate a DNA test utilizing Kompetitive Allele Specific PCR (KASP™) chemistry for identification of the number of copies of the soft scald and soggy breakdown HDI haplotype in *Malus*.

## Materials and methods

### Plant materials

A set of 132 unique apple accessions (e.g., cultivars, important breeding parents, selections, and unselected offspring) was used for candidate marker selection and marker validation. This set includes the following groups of individuals: one family (‘Honeycrisp’ × ‘MonArk’) of 20 unique individuals that have one or two copies of the HDI haplotype based on the results from Howard et al. (2018), seven individuals that represented unique breeding material from Washington State University, 67 UMN selections, and 38 important breeding parents used frequently in the UMN apple breeding program. Material was genotyped on the International RosBREED SNP Consortium 8K Illumina Infinium array v1 (Chagné et al. 2012) or the 20K Infinium SNP array (Bianco et al. 2014) and processed as described in Vanderzande et al. (2019) and SNP data was phased using FlexQTL™ (https://www.wur.nl/en/show/FlexQTL.htm) software. The germplasm utilized for marker evaluation were characterized for the number of HDI haplotypes using the methods described in Howard et al. (2018).

### Marker selection

Single nucleotide polymorphism (SNP) marker data from the International RosBREED SNP Consortium 8K Illumina Infinium array v1 (Chagné et al. 2012) and the 20K Infinium SNP array (Bianco et al. 2014) were used to identify potential DNA markers to assay. Physical marker positions reference the *Malus* × *domestica* GDDH13 v1.1 Whole Genome (Daccord et al. 2017). SNP markers near the QTL identified in Howard et al. (2018) ranged from positions 76,735 to 2,405,772 on LG2 and was explored for potential markers from both SNP arrays. Marker data was obtained for markers spanning from positions 31,061 to 5,036,446 on LG2 for test development. Investigation of SNP markers within the reported QTL positions had low marker-trait correlations, so the region investigated for potential markers for marker-trait correlation was extended in both directions up and down the linkage group. Additionally, ‘Honeycrisp’ had to be homozygous for each SNP marker considered for testing.

KASP™ primers were ordered according to LGC (https://biosearch-cdn.azureedge.net/assetsv6/assay-design-factsheet.pdf) requirements or were designed utilizing the IDT OligoAnalyzer tool (https://www.idtdna.com/calc/analyzer) with the following guidelines: GC content between 30 and 55%, melting point of 62 to 66 °C, 21 to 30 base pairs in length, a product of 50 to 100 base pairs, no more than 4 di-nucleotides, and no more than 3 G’s and/or C’s in the last 5 base pairs. FAM or HEX tag sequences were added to the 5′ end of the designed primer (Patterson et al. 2017). The primer sequences were delivered dry and rehydrated according to the resuspension calculator provided by IDT (https://www.idtdna.com/Calc/resuspension/) following the method of Patterson et al. (2017).

### DNA extraction and standardization

Young leaf tissue was collected, frozen at − 80 °C, lyophilized for 48 h, and stored on silica. A silica bead method was utilized to extract DNA. Extraction followed the procedure described by Edge-Garza et al. (2014), with additional grinding of leaf tissue when the initial grinding was insufficient. After extracted DNA was dried, it was rehydrated with deionized (DI) water. DNA content was determined using the nucleic acid setting on a NanoDrop 2000c spectrophotometer (Thermo Scientific) and when DNA concentrations were determined to be above 50 µg/µL, samples were reduced to approximately 50 µg/µL.

### KASP™ marker testing

KASP™ marker testing was performed according to the “KASP™ genotyping chemistry User guide and manual” provided by LGC (www.lgcgenomics.com). The wet DNA method was utilized; 5µL of DNA, 5µL of master mix, and 0.14µL of the primer mix were combined in each well of a 96-well plate. Six wells per plate utilized deionized water instead of DNA to serve as no-test controls and to avoid sample failure from poor plate sealing. PCR was conducted according to the suggested thermal cycling conditions provided by LGC on a C1000 Thermal Cycler (Bio-Rad) with a CFX96 Real-Time System plate reader (Bio-Rad).

An additional step of re-cycling (20 s at 94 °C, 1 min at 57 °C, repeated 3 times, followed by 30 s at 37 °C followed by the plate read) was performed after the first marker calls were made at the end of the PCR protocol. When the initial protocol failed to produce three distinct segregating groups, recycling was performed immediately to determine if additional cycles improved clustering. Markers were run as two or more technical replicates to verify consistency of marker performance.

### Marker evaluation

Automatic calling by CFX Manager 3.1 (Bio-Rad) was utilized for calling allele states for each marker. If individuals did not match the curated allele calling in Howard et al. (2018), the clustering results were visually inspected to identify errors in automated calling. Markers with poor results after manual inspection were not evaluated further.

Allele states according to the KASP™ assay were compared to expectations based on the known number of HDI haplotypes per genotype as previously reported in Howard et al. (2018). ‘Honeycrisp’ was utilized for each marker to determine which cluster corresponded to the individuals that had two HDI haplotypes.

Each individual was considered correctly characterized if its cluster group matched the expected genotype reported in Howard et al. (2018). Each group of individuals (zero, one, or two copies of the HDI haplotype) was evaluated for percent accuracy within each group, as well as across all groups. Markers with the highest accuracies were selected as the best candidates for a DNA test.

## Results

The initial pool for potential markers consisted of 143 markers originating from the International RosBREED SNP Consortium 8K Illumina Infinium array v1 and the 20K Infinium SNP array (Chagné et al. 2012; Bianco et al. 2014). These markers lie between positions 31,061 and 5,036,446 on LG2 (Online Resource 1).

Marker-HDI haplotype correlations were validated using all 132 accessions. Thirty-seven of these individuals had zero copies of the HDI haplotype, 74 individuals had one copy, and 21 individuals had two copies as characterized in or determined by using methods described in Howard et al. (2018) (Online Resource 2).

Sixteen SNP markers were screened for the development of this test. Three markers consistently failed to produce three clustering groups and were removed from further analysis. One marker characterized ‘Honeycrisp’ as heterozygous and was removed from further analysis. Twelve markers had reproducible marker calls (Online Resource 1). Prediction accuracies averaged over all three haplotype copy groups for these 12 markers ranged from 43 to 88% (Online Resource 2). The two markers with the highest prediction accuracies (Tables 1 and 2 and Online Resource 2) were effective individually on seedlings in the UMN apple breeding program.

**Table 1:** Accuracies of the two best performing DNA markers indicating the number of HDI copies (0, 1, or 2) an individual has based on Howard et al. (2018). Overall accuracy accounts for all individuals utilized for marker validation. Sample sizes for each group (*n*) indicate the number of individuals used to test each marker, respectively

		SNP_FB_0452022	SNP_FB_0949693
No. of HDI copies	2 (*n* = 21, 21)	86%	100%
	1 (*n* = 70, 71)	89%	79%
	0 (*n* = 34, 35)	88%	89%
Overall (*n* = 125, 127)		88%	85%

**Table 2:** Primer sequences for the two markers with the highest prediction accuracies. Primer sequences for the two forward components do not contain sequence tags for FAM and HEX fluorophores, which are added to the 5′ end of the primer; HEX or FAM may be assigned to either Forward 1 or Forward 2. FAM sequence tag is GAAGGTGACCAAGTTCATGCT and HEX sequence tag is GAAGGTCGGAGTCAACGGATT

Marker name	Marker component	Sequence (5′ to 3′)
SNP_FB_0452022	Forward 1	GTCTATTCTTTCAACCGAGAAAGTTTTGA
	Forward 2	CTATTCTTTCAACCGAGAAAGTTTTGC
	Reverse	CAACAAATAAAGTATTTCTCTTAAGCCTAT
SNP_FB_0949693	Forward 1	TCACTACAATTCTTCTCACTGCATGT
	Forward 2	CACTACAATTCTTCTCACTGCATGC
	Reverse	TACAACTAGTGAYGGGACATTCTCTAATAA

Three years of marker-assisted seedling selection have been performed at the UMN apple breeding program utilizing the two best performing markers (SNP_FB_0452022 and SNP_FB_0949693; Tables 1 and 2). In the first year, both markers characterized individuals into the same HDI haplotype copy number groups. Therefore, one marker (SNP_FB_0949693) was used for culling seedlings with two HDI haplotypes in subsequent years.

## Discussion

### DNA test successfully developed for soft scald/soggy breakdown QTL on LG2

A DNA test to identify the number of HDI haplotypes at the LG2 soft scald QTL, previously characterized in Howard et al. (2018), was successfully developed utilizing KASP™ chemistry. The KASP™ assay was selected over Taqman™ and rhAmp™ for several reasons. First, the QTL mapping performed in Howard et al. (2018) utilized SNP markers from the International RosBREED SNP Consortium 8K Illumina Infinium array v1 (Chagné et al. 2012). The SNP markers on 8K array can be easily converted to a KASP™ assay because both platforms utilize 50nt probe sequences for SNP markers. Secondly, KASP™ assays can be used with simple DNA extraction methods, can be done in-house or outsourced, and are relatively cost effective compared to other genotyping platforms (Majeed et al. 2019). SNP markers with the highest in silico correlations with the HDI haplotype were selected for validation. This DNA test has been deployed in the UMN apple breeding program to cull hundreds of seedlings predicted to carry two HDI copies. The results of this study demonstrate the potential utility of this DNA test to develop breeding populations with low relative soft scald and soggy breakdown incidence.

### Markers within or near the soft scald candidate gene region

The candidate gene for soft scald and soggy breakdown, *MdAAT1* (MDP0000637737), is associated with high levels of hexanol when suppressed (Souleyre et al. 2014). High levels of internal hexanol or the injection of fruit with hexanol have been associated with increased incidence and risk of soft scald formation (Wills and Scott 1970; Wills 1972, 1973; Leisso et al. 2016). A SNP marker located within the candidate region (ss475876983) is located in an exon for the *MdAAT1* gene but was not correlated with the HDI haplotype. Additional markers could be examined using the Axiom Apple 480K SNP genotyping array (Bianco et al. 2016), but this would require many unique cultivars to be genotyped, including ‘Honeycrisp.’ Marker data were not available from the 480K array for a wide set of germplasm that has been characterized for soft scald and soggy breakdown incidence rates or diplotype states at the LG2 QTL. The markers on the 480K array in the *MdAAT1* gene region should be examined in future studies; however, a SNP array might not capture all types of genetic variations (such as an insertion-deletion) so a targeted sequencing study may be a promising approach to identify higher marker-trait correlation. Additionally, other soft scald QTLs or candidate causal genes should be explored in future studies. An RNA-seq study could possibly identify additional candidate genes.

### Challenges

Several SNP markers demonstrated inconsistent results. Inconsistency could have been due to (1) primer sequences binding to multiple sites across the genome, probing multiple sites instead of only the desired site; (2) null alleles, where the primer sequence was absent in an individual’s genome, resulting in no or incomplete marker states being determined; or (3) null alleles or unclear allele calls due to the presence of secondary polymorphisms within the target genomic sequence.

One UMN selection was determined to have a deletion in the top portion of LG2 according to SNP array marker data. Additionally, soft scald and soggy breakdown incidence in this individual was documented; thus, the association between this individual’s haplotypes and its phenotype was not validated. While this was not commonly observed, use of this individual as a breeding parent would have implications for the use of this DNA test. Breeders should screen parents before utilizing this test on their offspring to verify marker prediction. Unique germplasm sets might contain individuals that have indistinguishable haplotypes from the HDI haplotype for the markers used in this study and these SNPs might not always differentiate other haplotypes that were not represented in this study.

### Breeding applications and considerations for soft scald LG2 QTL DNA test

The DNA test developed in this study could be used for parent and/or seedling selection (Table 3). Prior to use in seedling selection, it is recommended to evaluate the test on the parents to determine the expected segregation ratios and to compare them to the expected ratios based upon the characterization of the HDI haplotype in Howard et al. (2018). In addition, parental selection can be very cost effective, as parents only need to be screened a single time. Use of this DNA test in parental selection could allow breeders to make more informed crossing decisions that avoid crosses that might result in seedlings with two HDI haplotypes. Parent selection can be especially useful when making crosses to produce very large numbers of seedlings, as the cost of screening seedlings increases with the size of a cross, while the cost of screening parents is a one-time expense (Wannemuehler et al. 2019).

**Table 3:** Soft scald/soggy break down DNA test breeding considerations

Breeding consideration			Use soft scald/soggy breakdown DNA test			Other considerations
			Highly suggested	Suggested	Not critical	
Parent selection	Parent selection can be used to avoid crosses that could result in offspring homozygous for HDI haplotype	Yes	X			Test should be used for families where offspring might inherit two HDI haplotype copies
		No		X		Consider threshold of acceptance (one versus two copies in offspring) and adjust culling regime accordingly
Seedling selection	Breeding parent(s) typically exhibit high soft scald/soggy breakdown incidence and have at least one HDI haplotype	Yes	X			N/A
		No		X		
	Cultivar target market is non-stored product (e.g., consumer picked, immediate processing)	Yes			X	
		No	X			
	Postharvest storage options that minimize soft scald and soggy breakdown are readily available	Yes		X		
		No	X			

Several generations of crossing will result in marker-trait linkage deterioration. While breeding generations in apple are relatively long compared to many other crops, marker-trait linkage needs to be considered for long-term use. When considering new parents, pedigree information could be utilized to confirm expected diplotypes and to characterize parents with the markers suggested here. Additionally, marker-based seedling selection will no longer be required or effective due to allele fixation if a breeding program selects against the HDI haplotype and does not introduce germplasm into the breeding pool that carries an HDI haplotype.

### DNA testing in addition to phenotyping

While the utilization of marker-assisted selection could increase breeding efficiency, DNA tests will not eliminate the need for phenotyping. Although ‘Honeycrisp’ demonstrates high incidence of postharvest disorders, it has been commercially successful due to its unique texture. The most recent release from the UMN, ‘MN55,’ follows suit in that it has exceptional fruit quality but also exhibits high incidence rates of postharvest disorders. DNA tests could complement phenotyping for postharvest traits and predict which individuals might require more extensive fruit handling and postharvest protocols.

A breeder should be mindful that while seedlings could be screened and culled for highly undesirable alleles using DNA tests, diversity for other potentially valuable traits might be lost. Therefore, DNA tests should be used in concert with routine phenotyping.

Consideration of the target market and use of the cultivar should be considered prior to screening seedlings with a DNA test for soft scald and soggy breakdown. If the cultivar is not intended for long-term storage, screening for soft scald and soggy breakdown might not be relevant (Table 3). Although cultivars intended for long-term storage might still be highly successful despite having challenges with soft scald and soggy breakdown, as has been demonstrated with ‘Honeycrisp,’ they must command a premium price to compensate producers for additional costs and risk. Selection against the HDI haplotype for soft scald and soggy breakdown using these markers should mitigate these costs and risks in future cultivars.

## Conclusion

A DNA test utilizing KASP™ chemistry was developed to identify the number of HDI haplotypes for soft scald and soggy breakdown storage disorders in apple. Use of this test reduces the chances of selecting individuals that exhibit high incidence of soft scald and soggy breakdown postharvest disorders.

## Supplementary Information

Below is the link to the electronic supplementary material.

Supplementary file1 (XLSX 12 KB)

Supplementary file2 (XLSX 21 KB)

## Data Availability

Relevant data are included in this paper and its associated Online Resources.

## Code availability

NA.
